# Ubiquitin Dynamics in Complexes Reveal Molecular Recognition Mechanisms Beyond Induced Fit and Conformational Selection

**DOI:** 10.1371/journal.pcbi.1002704

**Published:** 2012-10-04

**Authors:** Jan H. Peters, Bert L. de Groot

**Affiliations:** Computational Biomolecular Dynamics Group, Max Planck Institute for Biophysical Chemistry, Göttingen, Germany; Stanford University, United States of America

## Abstract

Protein-protein interactions play an important role in all biological processes. However, the principles underlying these interactions are only beginning to be understood. Ubiquitin is a small signalling protein that is covalently attached to different proteins to mark them for degradation, regulate transport and other functions. As such, it interacts with and is recognised by a multitude of other proteins. We have conducted molecular dynamics simulations of ubiquitin in complex with 11 different binding partners on a microsecond timescale and compared them with ensembles of unbound ubiquitin to investigate the principles of their interaction and determine the influence of complex formation on the dynamic properties of this protein. Along the main mode of fluctuation of ubiquitin, binding in most cases reduces the conformational space available to ubiquitin to a subspace of that covered by unbound ubiquitin. This behaviour can be well explained using the model of conformational selection. For lower amplitude collective modes, a spectrum of zero to almost complete coverage of bound by unbound ensembles was observed. The significant differences between bound and unbound structures are exclusively situated at the binding interface. Overall, the findings correspond neither to a complete conformational selection nor induced fit scenario. Instead, we introduce a model of conformational restriction, extension and shift, which describes the full range of observed effects.

## Author Summary

Due to their importance in biological processes, the investigation of protein-protein interactions is of great interest. Experimental structures of protein complexes provide a wealth of information but are limited to a static picture of bound proteins. Ubiquitin is a signalling protein that interacts with a wide variety of different binding partners. We have used molecular dynamics simulations to compare the dynamic behaviour of bound and unbound ubiquitin in complex with different binding partners. Our observations suggest that the conformations accessible to bound ubiquitin, while often restricted in comparison to unbound ubiquitin, still occupy a subspace of the conformational space as those of unbound ubiquitin. This corresponds to the “conformational selection” binding model. Only on a local level near the binding interface, differences between bound and unbound structures were found in specific regions of the bound ensemble. To account for the different types of behaviour observed, we extend the currently available binding models by distinguishing conformational restriction, extension and shift in the description of binding effects on conformational ensembles.

## Introduction

Protein-protein interactions are crucial in most biological processes, yet the principles governing the conformational effects of these interactions are still poorly understood. X-ray structures of protein complexes provide a wealth of high resolution structural information but reflect a static snapshot of the structure, leaving the mechanism of complex formation and dynamics in the complex unaddressed. In addition, compared with the growing number of experimentally determined structures of unbound proteins, there is only a small number of known structures of protein complexes. Computational methods are being developed to derive complex conformations from unbound structures, but this remains a challenging and highly non-trivial task [1]. With the increase in computational power, flexibility has been introduced in the computational methods, and shows promising results [2].

Two different models have been suggested to explain the conformational differences observed experimentally between bound and unbound proteins. The induced fit model [3] postulates that after the formation of a preliminary “encounter complex”, the interaction between the binding partners induces conformational changes into the complex structures. The conformational selection model [4]–[6] takes into consideration the inherent flexibility of proteins. According to this model, unbound proteins can with a certain probability sample the same conformations as observed when bound. In this model, changes in the free energy landscape of the protein due to interactions in the complex shift the conformational density towards the complex structure upon binding. More recent studies [7], [8] have indicated that elements of both models play a role in protein binding with an initial conformational selection step followed by induced fit rearrangements [9].

A good model system to investigate the conformational effects of complex formation is ubiquitin with its binding partners. Ubiquitin is a 76 residue protein that plays an important role in metabolic pathways, as the ubiquitination (covalent attachment of ubiquitin to a lysine side chain of a protein) can, among other functions, control the degradation or regulate transport of this protein. In this function, ubiquitin is recognised by and interacts with a multitude of other proteins. Lange et al. [10] found evidence for conformational selection, showing low root mean square (rms) differences between NMR solution structures of isolated ubiquitin and x-ray structures of ubiquitin in complexes. Wlodarski and Zagrovic [8] found indications for “residual induced fit” by performing statistical analysis on the atomic detail of the same structures. It has recently been shown [11] however, that the observed differences between the experimental bound structures and a molecular dynamics (MD) ensemble of unbound ubiquitin decrease with an increasing number of snapshots considered from the simulation ensemble, indicating that indeed conformational selection largely suffices to explaining the conformational heterogeneity of ubiquitin in different complexes.

Thus far most studies have focused on static snapshots of ubiquitin complexes in comparison to solution ensemble of unbound ubiquitin. Here, based on several experimental structures of ubiquitin in different complexes [12]–[22], we have performed and analysed MD simulations of ubiquitin interacting with different binding partners, thereby finally taking into account the flexibility the proteins display in the bound state. It has been shown [23] that MD simulations of unbound ubiquitin agree quantitatively with solution NMR data.

Statistical evaluation of simulations of ubiquitin both in the presence and the absence of a binding partner indicates conformational selection to be the appropriate model for complex formation when considering the dominant backbone dynamics, while some localised differences between bound and unbound ensembles can be found near the binding interface.

## Results

Seventeen structures of ubiquitin in complex with eleven different binding partners were selected from the protein database (PDB) [24] (see table 1 for PDB codes and references). The complexes were selected from the structures available in the PDB according to quality and structural variety of ubiquitin. Each of these structures was simulated both in the presence of the binding partner (bound) and in its absence (control). Additional simulations starting from two x-ray structures without binding partner (1UBI [25] and 1UBQ [26]) were conducted for comparison.

**Table 1:** Structures used for simulation setup.

PDB code	binding partner	reference
1NBF	Ubiquitin carboxyl-terminal hydrolase 7 (HAUSP)	[12]
1P3Q	CUE domain of Vacuolar protein sorting associated protein (Vps9p)	[13]
1S1Q	Tumor susceptibility gene 101 protein (TSG101)	[14]
1UBI	none (unbound reference)	[25]
1UBQ	none (unbound reference)	[26]
1UZX	UEV domain of Vps23	[15]
1XD3	Ubiquitin Carboxyl-terminal esterase L3 (UCH-L3)	[16]
2D3G	UIM from hepatocyte growth factor-regulated tyrosine kinase substrate (Hrs-UIM)	[17]
2FIF	Rab5 GDP/GTP exchange factor	[18]
2G45	Ubiquitin carboxyl-terminal hydrolase 5	[19]
2HTH	Vacuolar protein sorting protein 36	[20]
2IBI	Ubiquitin carboxyl-terminal hydrolase 2	[21]
2OOB	E3 ubiquitin-protein ligase CBL-B	[22]

Structures used for simulation setup.

### Conformational overlap and restriction observed in the main modes of ubiquitin backbone dynamics

**Figure 1:**
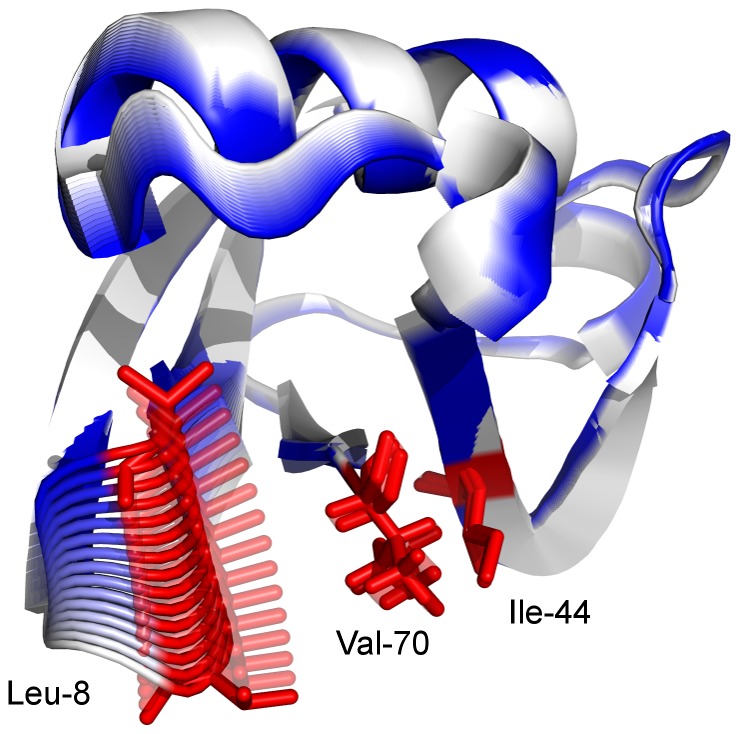
Visualisation of the first PCA eigenvector. It corresponds to pincer mode already described in [10]. The residues of the hydrophobic patch (Leu8, Ile44 and Val70) are marked in red.

**Figure 2:**
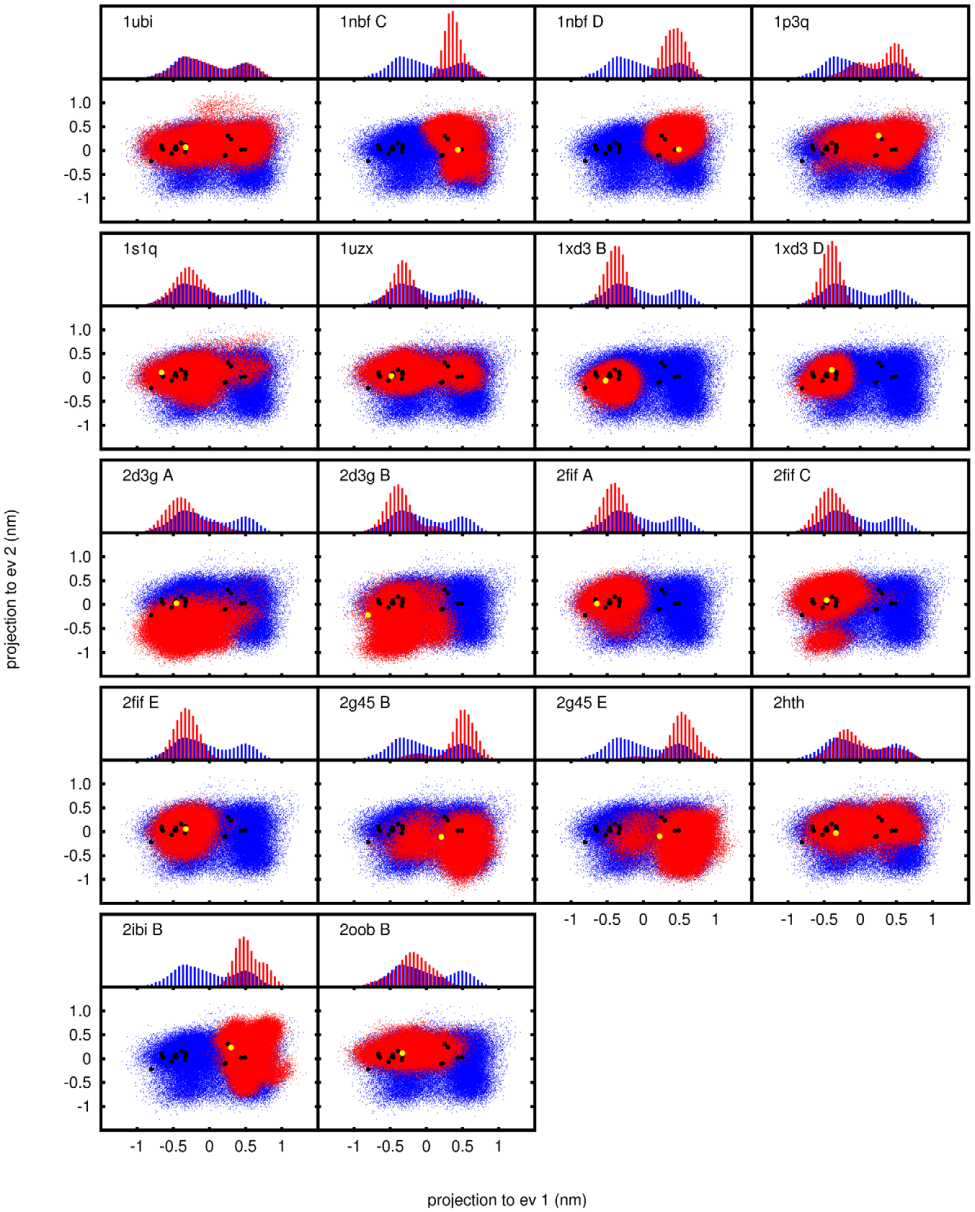
PCA results. Projection to the first two PCA-eigenvectors based on the backbone of residues 1–70 of all simulated ensembles. For comparison, the unbound reference ensemble is also plotted in blue. The original xray structures are marked in yellow. Histograms for the projection on the first eigenvectors are plotted above the corresponding plots. PDB codes for the starting structures of the simulations are in the upper left corner of each plot. Capital letters denote the chain identifier.

To investigate the effect of binding on the backbone dynamics of ubiquitin, a principal component analysis (PCA) of the backbone atoms of residues 1–70 of the ubiquitin chain was performed. It reveals a functionally relevant “pincer mode” in the first eigenvector (Figure 1, previously described in [10]), that has direct influence on the geometry of the “hydrophobic patch”, a group of three hydrophobic residues (Leu 8, Ile 44 and Val 70) that are involved in most binding interfaces of ubiquitin with other proteins (Figure S1). A 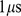 simulation of unbound ubiquitin (Figure 2 “1ubi”) spans a conformational space similar to that covered by a large number of known experimental structures from both X-ray and NMR experiments (see also Figure S2).

Like the unbound simulation ensemble, also simulations of bound ubiquitin show considerable conformational variety and in fact show a conformational entropy similar to unbound simulations (Figure S3, estimated according to [27]).

However, while the dynamics of bound ubiquitin ensembles are considerable, specific restrictions can be observed in most of the 11 complexes when considering the main backbone dynamic modes (Figure 2). All bound trajectories sample a subspace of that spanned by the unbound trajectory. The first two eigenvectors displayed here cover about 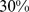 of the total variance (Figure S4), and are the only ones for which significant differences between bound and unbound ensembles could be observed (Figures 2, S5, S6, S7).

In all but one of the bound ensembles, the free energy profile along the “pincer mode” appears to have changed to shift the equilibrium towards either side of the conformational range (Figure 2). While in most cases the shift is partial and most of the conformational space still is sampled (albeit with a lower probability on one side), some trajectories can be described as purely “open” (the ensembles based on the PDB structures 1xd3 and 2fif) or “closed” (ensembles based on PDB structures 1nbf and 2ibi). Besides the obvious exception of the ensemble 1ubi based on an unbound ubiquitin structure, only one ensemble of bound ubiquitin (2hth) shows a distribution very similar to the unbound reference ensemble and therefore does not indicate restriction of the ubiquitin dynamics in the complex.

**Figure 3:**
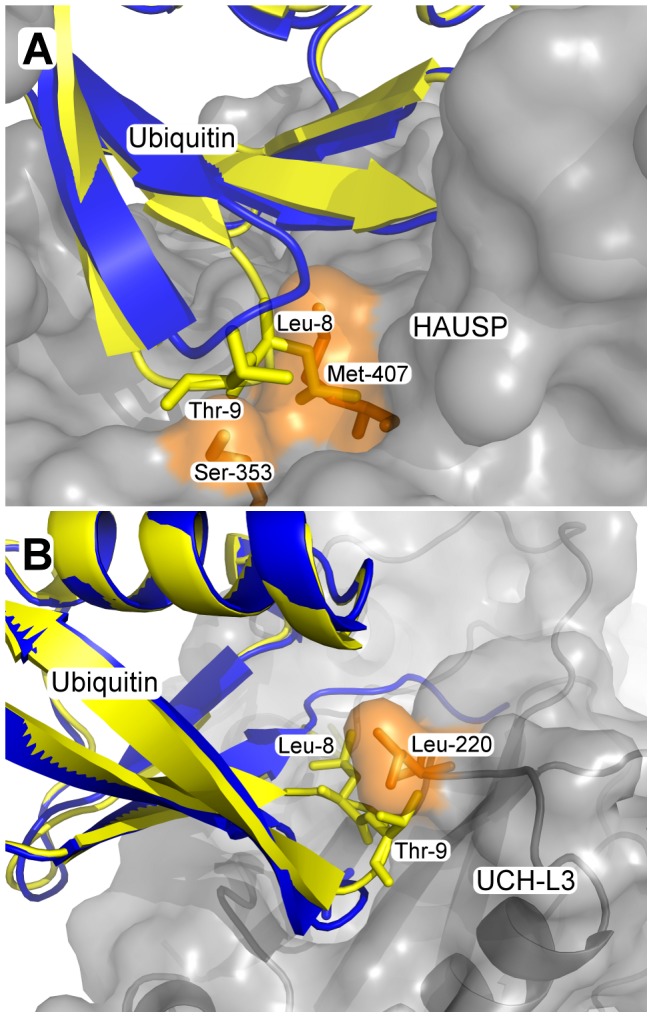
Steric clashes restricting pincer mode dynamics in the complex. Detail from the xray structure (A) 1nbf (ubiquitin bound to HAUSP) and (B) 1xd3 (ubiquitin bound to UCH-L3). For each structure, the compatible ubiquitin structure is shown in blue, while an incompatible structure that has been fitted to the same position is shown in yellow. Clashes with the binding partner are marked in orange.

Figure 3 shows a possible explanation for the restriction in both the open and closed states in two of the complexes. Ubiquitin bound to HAUSP (the binding partner in complex 1nbf) resides in a cavity that restricts its conformation in the closed state. In the open conformation, clashes would occur between residues Leu-8 and Thr-9 of ubiquitin and Ser-353 and Met-407 of HAUSP. In the complex of ubiquitin and UCH-L3 (complex 1xd3), residues Leu-8 and Thr-9 reside in a cavity of UCH-L3 when ubiquitin is in the open conformation. In the closed conformation, a clash between these residues and Leu-220 of the binding partner would occur which precludes these conformations.

The C-terminal tail of ubiquitin, comprising residues 71–76, shows high flexibility in the unbound and most of the bound ensembles to a degree that some parts of it are fully resolved only in four of the eleven experimental structures used for simulation setup (PDB codes 1nbf, 1s1q, 1ubi and 2g45) with three experimental structures (PDB codes 1uzx, 1xd3 and 2ibi) missing only the last residue. Four of these structures (1nbf, 1xd3, 2g45 and 2ibi) are the only ones in this study that show a significantly stronger restriction of dynamics if the C-terminal residues are included in the analysis (Figure S8). Besides this, the dynamic behaviour of the ubiquitin tail seems to be rather unstructured. Hence, like in other studies [10], [11] we focus on the analysis of ubiquitin dynamics to residues 1–70 as we have done in the PCA and will do in the following analysis, where inclusion of the C-terminal residues also does not qualitatively change the results while significantly increasing estimated uncertainties (Figure S9).

### Differences between bound and unbound conformational ensembles as observed using Partial Least Squares Discrimination Analysis (PLS-DA)

The principal component analysis indicates conformational overlap between bound and unbound ensembles on the level of the dominant collective backbone degrees of freedom. However, PCA as a method is not aimed at discrimination, especially if the amplitude of the differences is small compared to the variation within the ensembles. It is well possible that differences between the ensembles on a more local level are not detected by PCA. To determine differences between multidimensional ensembles, partial least squares discrimination analysis (PLS-DA, cf. Materials and Methods) has been found to be more effective than PCA [28].

**Figure 4:**
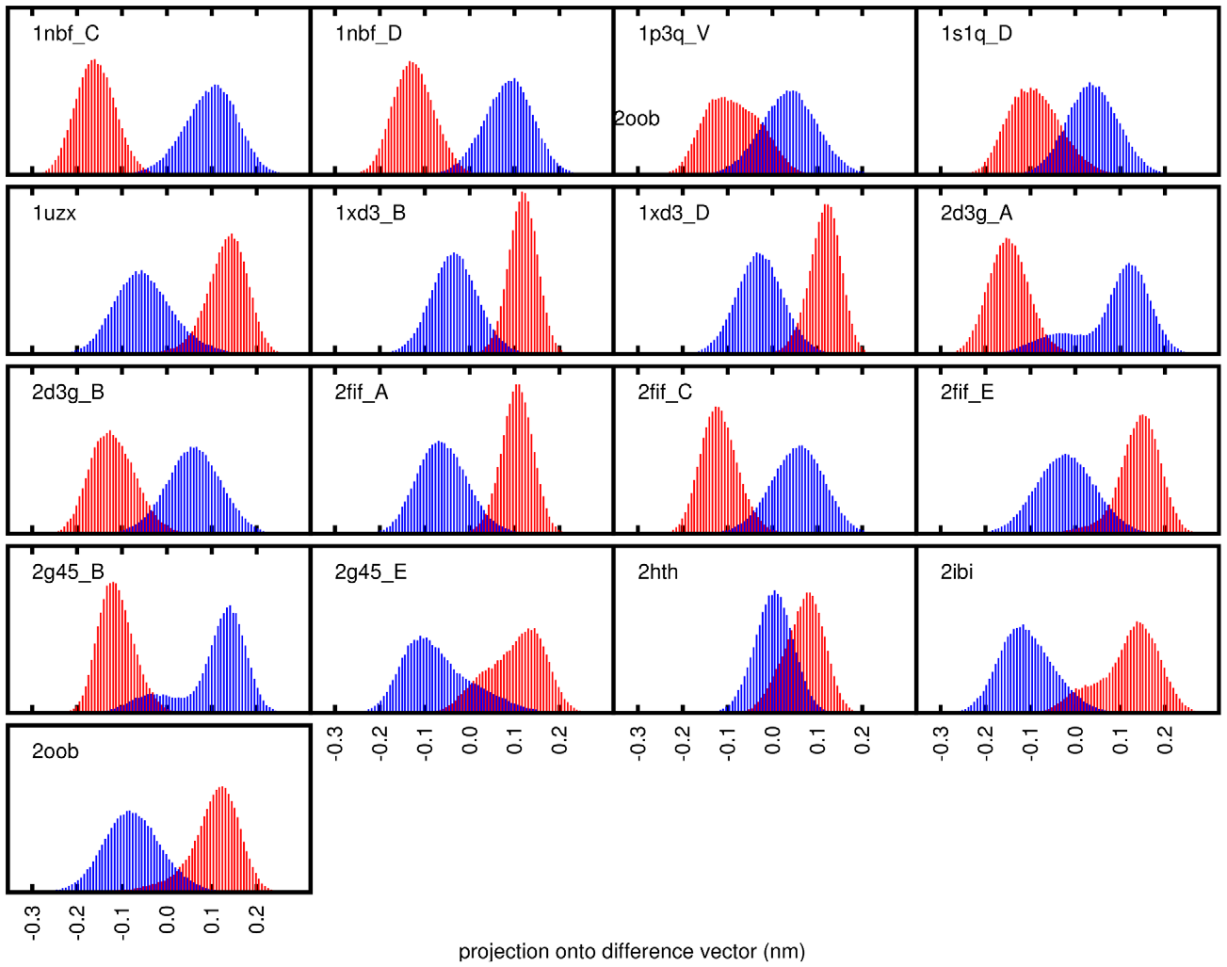
PLS-DA results on backbone atoms of residues 1–70. Different bound ensembles (red) and the unbound reference ensemble (blue) have been projected onto the difference vector between these ensembles as determined by PLS-DA.

Indeed, using this method, models can be found to almost completely distinguish some of the bound ensembles from the unbound reference ensemble The magnitude of these differences is however significantly smaller than that of the main fluctuation modes of ubiquitin (compare length-scales in Figures 2 and 4).

PLS-DA distinguishes between ensembles both on a global as well as on a local level. Even the systematic difference between two ensembles in e.g. a single side chain rotamer will result in a zero overlap.

**Figure 5:**
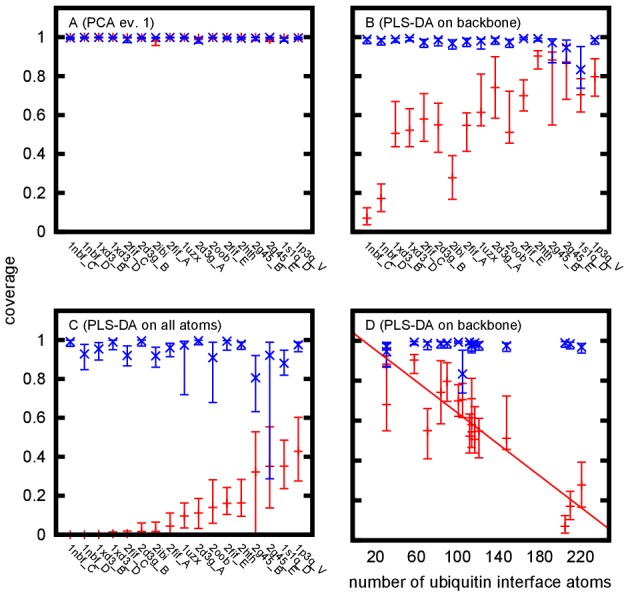
Coverage of different ensembles by the unbound reference ensemble. The histogram-coverage of bound ensembles (red) compared to coverage of unbound control ensembles (blue) after projection of the structures onto the first PCA-eigenvector (fig. 2) of backbone atoms of residues 1–70 (A), the PLS-DA difference vector of backbone atoms of residues 1–70 (B and D), and the PLS-DA difference vector of all non-hydrogen atoms of residues 1–70 (C). Ensembles in A–C have been sorted according to the coverages displayed in C. Uncertainties have been determined using the stationary bootstrap method.

While both bound and unbound control ensembles are fully covered by the unbound reference ensemble along the main mode of ubiquitin dynamics (Figure 5 A), the coverage of the bound ensembles after PLS-DA on the backbone atoms of residues 1–70 (Figure 5 B) is found to be significantly lower. When also considering all non-hydrogen side chain atoms (Figure 5 C), several bound ensembles are no longer covered by the unbound reference ensemble. To validate the significance of the observed differences, the same method has been applied to calculate the coverage of unbound control ensembles by the unbound reference ensemble. It was found to be significantly higher (i.e. almost full), as expected.

The observed differences correlate well (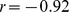) with number of ubiquitin atoms involved in binding (i.e. with a distance of less than 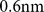 from the binding partner, Figure 5 D). Hence a more extensive binding interface correlates with more significant differences to the unbound state.

### Local conformational differences on the residue level can be observed using PLS-DA

To localise differences between bound and unbound ensembles, individual PLS-DA calculations were performed on the conformations of each residue (including side chains) of ubiquitin separately after fitting the backbone of the whole chain.

**Figure 6:**
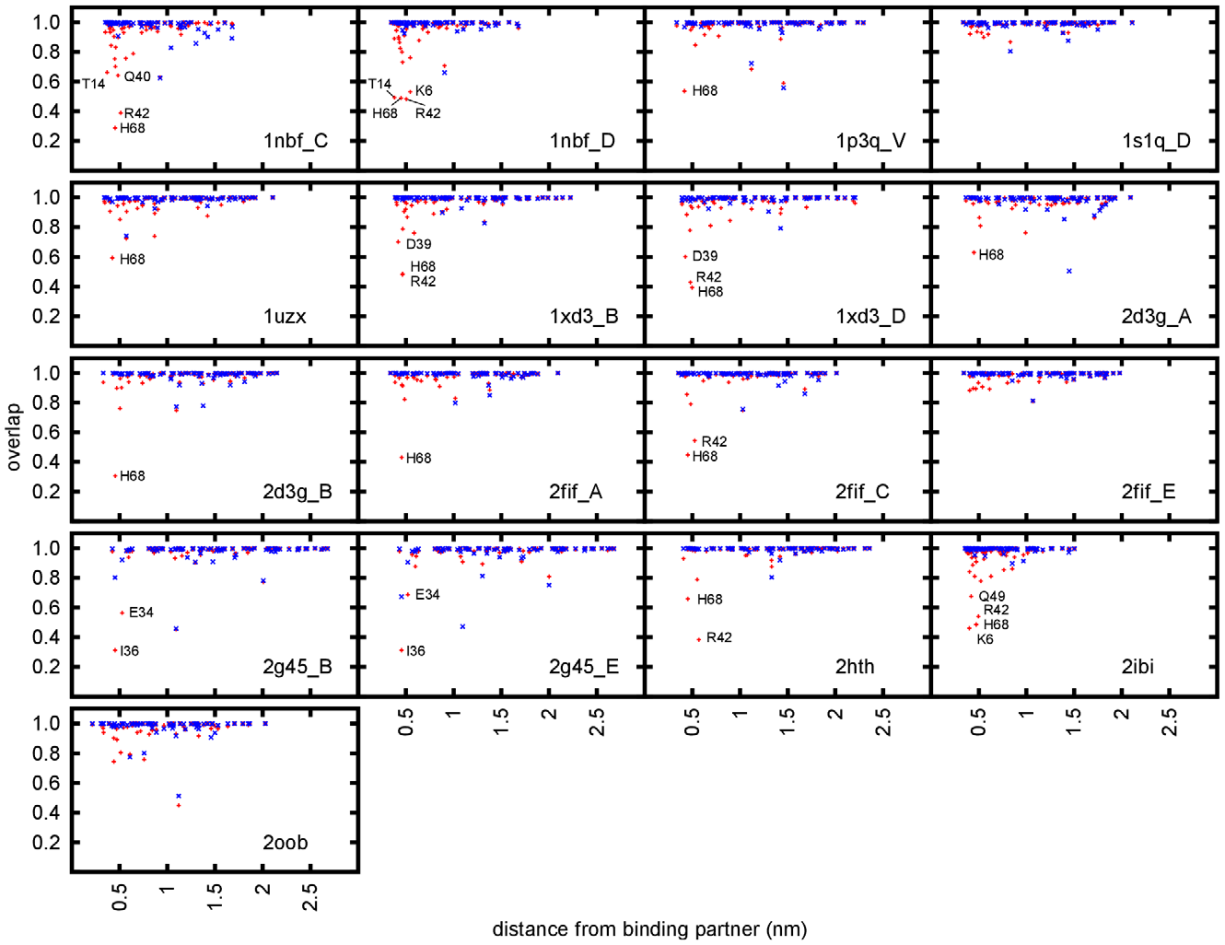
Overlap between bound (red) and unbound control (blue) ensembles. Overlap has been calculated with the unbound reference ensemble after projection to the difference vector found by PLS-DA on single residues after fitting to the backbone and plotted versus distance from the binding partner. Residues displaying a significant difference in the bound ensemble are labelled.

Only a small number of residues for each complex ensemble show an overlap with the unbound reference ensemble which is significantly below 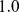 and none of them shows an overlap below 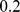, Most of the unbound control ensembles show almost complete (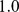) overlap with the reference ensemble. The observed differences due to binding interactions are local, as all of the residues found to change their conformation are located near the binding partner (Figure 6).

**Figure 7:**
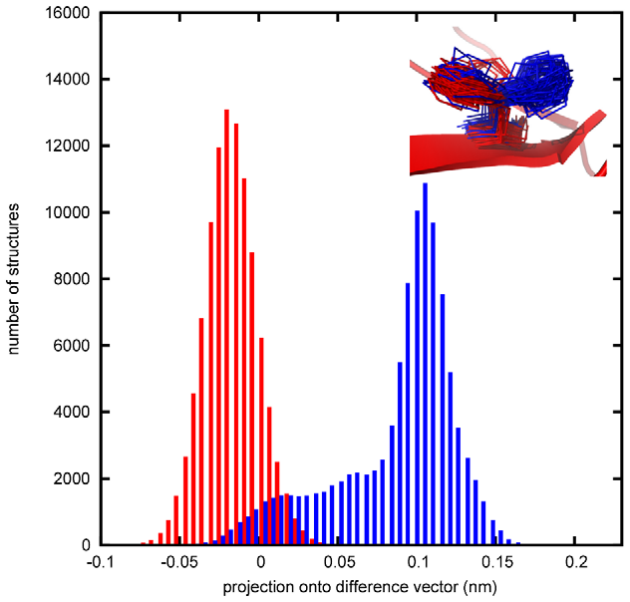
Example of a difference found in PLS-DA and it's structural origin. Histogram of the projection of bound (red) and unbound (blue) ensemble onto the difference vector found by PLS-DA for residue His68 of ensemble 1nbf chain C. Out of all 11 complexes studied, this residue shows the smallest overlap between bound and unbound ensembles. The inset shows the corresponding structures from the simulation ensembles.

Again, in none of the cases, a complete distinction between bound and unbound ensembles could be found. Even for the residue displaying the smallest overlap between bound and unbound ensembles (residue His68 in ensemble 1nbf chain C) a small fraction of bound structures can be found in the same conformational region as the unbound ones (Figure 7).

## Discussion

We compared ensembles of ubiquitin structures from molecular dynamics simulations with and without binding partners aimed at a detailed investigation of the conformational effects of protein binding.

The main collective mode of fluctuation found in unbound ubiquitin is the “pincer mode” which strongly influences the shape of the binding surface (Figure 1). It has been indicated [10] that the flexibility of this mode is essential for ubiquitin to interact with a large number of different binding partners. Indeed, this mode is characteristically affected by binding, as all but one of the bound ensembles show a significant shift or restriction of conformational density, while still the whole range of flexibility of unbound ubiquitin is required to accommodate all observed bound states. Since all bound ensembles are completely covered by the unbound ensemble along the pincer mode, the conformational selection model is applicable for this aspect of binding.

Employing the partial least squares discrimination analysis method, that specifically aims at identifying differences between ensembles, low amplitude difference modes between bound and unbound ubiquitin ensembles were identified.

The observation of the unbound protein displaying the bound state conformation is often considered indicative of conformational selection ([6], [10], [11], [29]). We observed a significant fraction of the unbound ubiquitin ensemble showing a strong similarity (especially in the main pincer mode) to the conformations of bound ubiquitin. This is consistent with a conformational selection binding scenario, while the differences between bound and unbound ensembles on the local level indicate residual induced fit effects as have been introduced in recent binding models [7]–[9].

It is still possible that a portion of the binding events occurs according to an induced fit scenario. An alternative classification of the binding process is based on the inclusion of binding kinetics [30]–[32]. As we have concentrated our analysis on the comparison of bound and unbound states rather than on association and dissociation events, a kinetic approach is beyond the scope of this paper.

An aspect not considered in recently discussed binding models [7]–[9] is the dynamic nature of bound proteins. Earlier work [33] already indicated that binding does not necessarily decrease the conformational entropy of proteins. We have also found that the dynamics of the bound ubiquitin ensembles are on a similar scale as those of unbound ubiquitin (Figures 2, S3).

**Figure 8:**
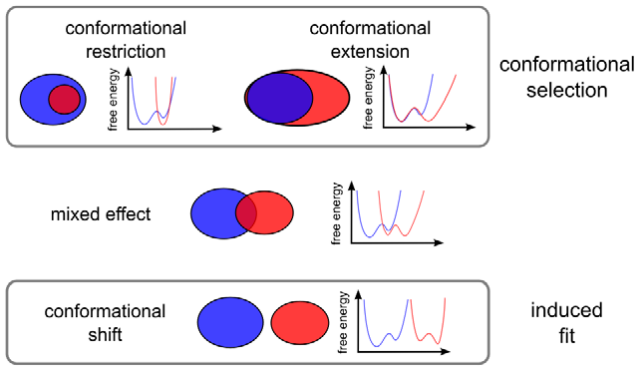
Schematic description of the proposed binding models. The blue ensemble would be that of the unbound protein, the red that of the bound. A sketch of possible free energy profiles fitting the corresponding models is given on the right.

In general, two effects of binding on the conformational space of the protein can be expected (Figure 8). Conformations accessible to the unbound protein can be prohibited by interactions (Figure 3) with the binding partner (conformational restriction) while conformations that were energetically unfavourable to the unbound protein can become accessible due to favourable interactions with the binding partner (conformational extension).

These two effects are not mutually exclusive and indeed in most cases we observe a combination of both effects in the binding behaviour of ubiquitin. In the most extreme cases, all conformations accessible to the unbound protein are restricted, with all the conformations in the complex being the effect of conformational extension. This “conformational shift” corresponds best to the induced fit binding model.

In the case of conformational extension, changes of the energy landscape due to binding allow the protein to access conformations that are energetically unfavourable in the absence of the binding partner. While not generally considered, conformational extension is well compatible with the conformational selection model of binding, as the binding process itself can well take place in the overlap between the bound and unbound states.

Most complexes considered in this study can be described by the scenario of conformational extension combined with conformational restriction, showing a significant overlap between bound and unbound ensembles. Interestingly, also for those complex with near-zero overall overlap, substantial overlap is found between the bound and unbound states on the level of individual residues. Hence, for these complexes, each residue samples states in the unbound state that are found in the bound state, but the probability to find all contact residues in a complex compatible state *simultaneously* approaches zero for these complexes, resulting in zero overall overlap.

The consideration of conformational ensembles is a common feature of modern computational protein docking approaches to account for conformational changes due to binding [2], [34]. Our results suggest that while native conformational ensembles are likely to yield good binding conformations on a global scale, small-scale structural adaptions at the binding interface seem to occur that are specifically caused by interactions with the binding partner.

## Materials and Methods

### Molecular dynamics simulation

From the Protein Data Bank (PDB, [24]), eleven structures of ubiquitin in complex with a binding partner and two structures of unbound ubiquitin were selected (see table 1 for PDB codes and references). To avoid unspecific interactions, structures containing more than one complex were separated before simulation. Simulations were performed using GROMACS 4 [35]. In accordance with recent evaluations of simulation setups ([23] and [36]) the ffamber port [37] of the amber99sb force field [38], particle-mesh Ewald electrostatics [39], [40] were employed with fourth order interpolation, a maximum grid spacing of 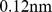 and a cutoff of 0.9 nm. Water was modelled using the SPC/E water model [41]. A twin-range van der Waals cut-off (0.9/1.4 nm) was used. Both protein and solvent where separately held at a temperature of 300 K using the v-rescale algorithm [42] (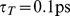) and pressure coupled at 1 bar using the Berendsen algorithm [43] (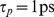). A 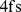 time step was achieved by using Lincs bond constraints [44], SETTLE [45] constraints on water and virtual sites [46]. After a steepest descent energy minimisation and a 1 ns equilibration using position restraints on the protein, 10 production runs of 100 ns each were performed for each ensemble, using random starting velocities. Simulation snapshots were taken every 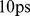 for analysis (this seems to be more than sufficient as a 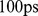 sampling returns about the same general results as can be seen in Figure S10). For each simulation of bound ubiquitin, an unbound control simulation from the same starting structure of ubiquitin was performed without the binding partner. To allow for relaxation of structural differences, the first 10 ns of the production run was not included in the analysis. An unbound reference ensemble was created from simulation trajectories based on the unbound x-ray structures 1UBI and 1UBQ and these unbound control trajectories. Ensembles based on similar structures (i.e. from starting structures from the same PDB entry) were not used in comparisons with either bound or control ensembles.

### Principal component analysis

Principal component analysis [47]–[49] has been performed on a structural ensemble consisting of structures (snapshots every 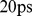) from the 17 bound and 20 unbound simulation ensembles simulated for this study. PCAs based on only unbound or only unbound simulation ensembles resulted in very similar eigenvectors (Figures S11 and S12). The backbone atoms of residues 1–70 of ubiquitin have been used for both fitting and analysis resulting in 630 degrees of freedom. All ensembles have been projected on the first eight eigenvectors found in this analysis (fig. 2, S5, S6, S7).

### Partial Least Squares Discrimination Analysis

Partial least squares regression (PLS) can be used to find a linear model to calculate an external parameter from protein structures. By defining a label of which structures belongs to which class (in this case 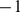 denoting structures from unbound ensembles and 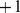 denoting structures from bound ones) as this external parameter, PLS can be used to calculate a model which describes differences between these two classes of structures provided such a difference exists. The resulting linear model yields a difference vector similar to a PCA eigenvector.

If a structural difference between the classes exist, the projection of structures onto this difference vector will make it possible to assign a structure to one or the other class. If it is not possible to completely distinguish structures belonging to the two different classes, the model will still produce the best possible distinction, allowing quantification of the remaining overlap between bound and unbound ensembles. For this, both ensembles are projected onto the difference vector and histograms of the projections are calculated (fig. 4).

The PLS-DA algorithm used in this study produces a model that maximised the difference of the projection of two structures from different classes (bound vs. unbound) while minimising the difference between structures from the same class. Consequently, if more than one structural mode can be used to distinguish the two classes, the resulting model will not necessarily represent both of them, especially if one would result in stronger variation within the classes. While the method can be used to determine whether or not a full distinction between bound and unbound ensembles can be found, additional steps are necessary to fully characterise the structural differences. For this, PLS-DA was performed on sub-groups of atoms (i.e. the backbone as well as each residue including side chain individually) after fitting of the ensemble on the backbone atoms.

Helland's Algorithm [50] as implemented by Denham [51] was used to perform the partial least squares discrimination analysis (PLS-DA) on the simulation ensembles. PLS performs a regression on a basis that is optimised to correlate with the external parameter. Choosing a high dimensional basis generally improves the quality of the model on the training data but can decrease its predictive power due to overfitting. For this, the combined structures of the bound and unbound ensemble were divided into a model building set (containing half of both ensembles) and a test set (containing the other half of each ensemble). Comparing model quality for both training and test set (Figure S13) shows both correlations to reach a plateau for 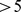 dimensions and no overfitting effects, so a ten dimensional basis was used in all PLS-DA calculations.

For comparison, both ensembles were sorted into the same set of 100 bins spanning their combined range. The overlap of one ensemble by the other is defined as the normalised sum of the products of the number of structures for each bin. Coverage of one ensemble by another is defined as the fraction of structures from the first ensemble in bins containing a minimum number (50) of structures from the other ensemble.

The stationary bootstrap algorithm [52] was used to estimate the uncertainty of overlaps and coverage.

## Supporting Information

Figure S1
**The importance of the hydrophobic patch in ubiquitin binding.** Distance of the ubiquitin residues in all complexes from the binding partner. Residues Leu-8, Ile-44 and Val-70 (the “hydrophobic patch) have been marked in red. With two exceptions (Ile-44 and Val70 in complex 2g45) all hydrophobic patch residues are within 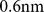 of the binding partner.(TIFF)

Figure S2 **Simulation ensembles cover the same conformational space as known experimental structures.** PCA projection of unbound MD simulation (starting structure 1ubi, red and a collection of experimental xray (black, 139 structures from 63 different PDB entries) and NMR (blue, 783 structures from 35 different PDB entries) structures.
(TIFF)

Figure S3 **Bound ensembles show significant structural dynamics.** Conformational entropy observed in unbound (blue) and bound (red) simulation ensembles estimated according to the Schlitter formula excluding (A) and including (B) the flexible C-terminus.
(TIFF)

Figure S4 **Eigenvalue spectrum for the first 50 eigenvectors of the PCA used in the main paper (backbone atoms 1–70).**
(TIFF)

Figure S5 **Projection to higher order eigenvectors shows no significant differences between bound and unbound ensembles.** Projection to PCA-eigenvectors 3 and 4 of all simulated bound ensembles based on the backbone of ubiquitin residues 1–70. For comparison, the unbound reference ensemble is also plotted in blue, the original xray structures are marked in yellow. PDB codes for the starting structures of the simulations are in the upper right corner of each plot. Capital letters denote the chain identifier.
(TIFF)

Figure S6 **Projection to higher order eigenvectors shows no significant differences between bound and unbound ensembles.** Projection to PCA-eigenvectors 5 and 6 of all simulated bound ensembles based on the backbone of ubiquitin residues 1–70. For comparison, the unbound reference ensemble is also plotted in blue, the original xray structures are marked in yellow. PDB codes for the starting structures of the simulations are in the lower left corner of each plot. Capital letters denote the chain identifier.
(TIFF)

Figure S7 **Projection to higher order eigenvectors shows no significant differences between bound and unbound ensembles.** Projection to PCA-eigenvectors 7 and 8 of all simulated bound ensembles based on the backbone of ubiquitin residues 1–70. For comparison, the unbound reference ensemble is also plotted in blue, the original xray structures are marked in yellow. PDB codes for the starting structures of the simulations are in the lower left corner of each plot. Capital letters denote the chain identifier.
(TIFF)

Figure S8 **Alternative PCA including all backbone atoms of ubiquitin.** Projection to the first two PCA-eigenvectors of all simulated bound ensembles based on the backbone of all ubiquitin residues (1–76). For comparison, the unbound reference ensemble is also plotted in blue, the original xray structures are marked in yellow. Histograms for the projection on the first eigenvectors are plotted above the corresponding plots. PDB codes for the starting structures of the simulations are in the upper left corner. Capital letters denote the chain identifier.
(TIFF)

Figure S9 **Coverage of different ensembles by the unbound reference ensemble.** The histogram-coverage of bound ensembles (red) compared to coverage of unbound control ensembles (blue) after projection of the structures onto the first PCA-eigenvector (fig. 2) of backbone atoms of residues 1–76 (A), the PLS-DA difference vector of backbone atoms of residues 1–76 (B and D), and the PLS-DA difference vector of all non-hydrogen atoms of residues 1–76. Ensembles in A–C have been sorted according to the coverages displayed in C. Uncertainties have been determined using the stationary bootstrap method.
(TIFF)

Figure S10
**Influence of sampling on ensemble coverage.** Comparison of the results illustrated in 5 B (here: A) and C (here: B) for two different sampling frequencies, 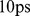 (green) and 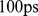 (orange).(TIFF)

Figure S11 **Comparison of PCA eigenvectors based on all trajectories and unbound trajectories only.** Inner product calculated between the first 10 eigenvectors of both PCAs.
(TIFF)

Figure S12 **Comparison of PCA eigenvectors based on all trajectories and bound trajectories only.** Inner product calculated between the first 10 eigenvectors of both PCAs.
(TIFF)

Figure S13 **Cross correlation test of PLS-DA models.** Correlation between target and model for training (green) and test (orange) set for PLS-DA between unbound and bound ensembles based on backbone atoms of residues 1–70 evaluated for different basis dimensionality.
(TIFF)
