# Supplementary figures and images for: Ubiquitin Dynamics in Complexes Reveal Molecular Recognition Mechanisms Beyond Induced Fit and Conformational Selection
Source: PLoS Comput Biol. 2012 Oct 4;8(10):e1002704. doi: 10.1371/journal.pcbi.1002704 (PMC3464204; doi:10.1371/journal.pcbi.1002704)

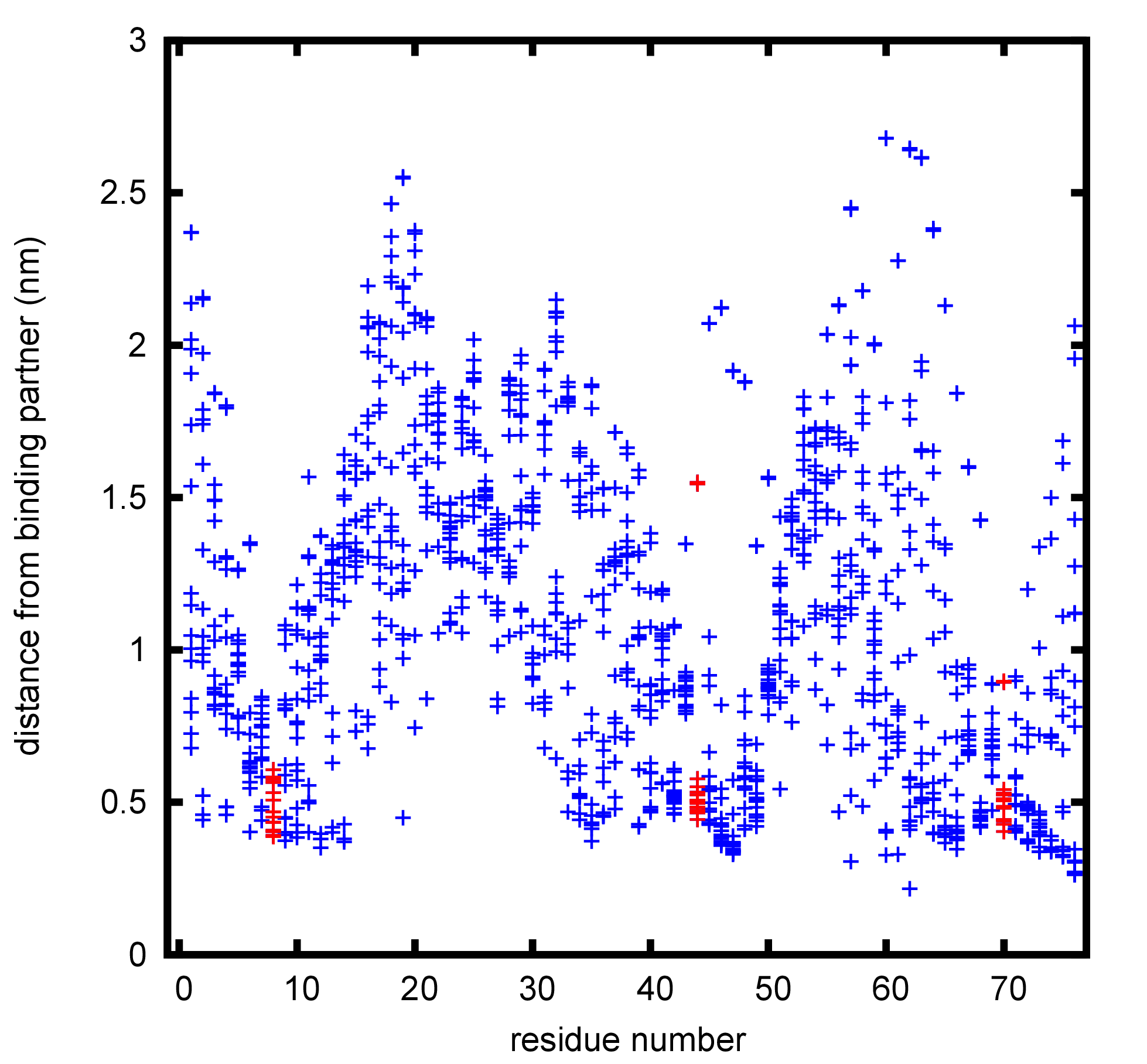

Supplement: Figure S1 — The importance of the hydrophobic patch in ubiquitin binding. Distance of the ubiquitin residues in all complexes from the binding partner. Residues Leu-8, Ile-44 and Val-70 (the “hydrophobic patch) have been marked in red. With two exceptions (Ile-44 and Val70 in complex 2g45) all hydrophobic patch residues are within of the binding partner. (TIFF) [file pcbi.1002704.s001.tif]

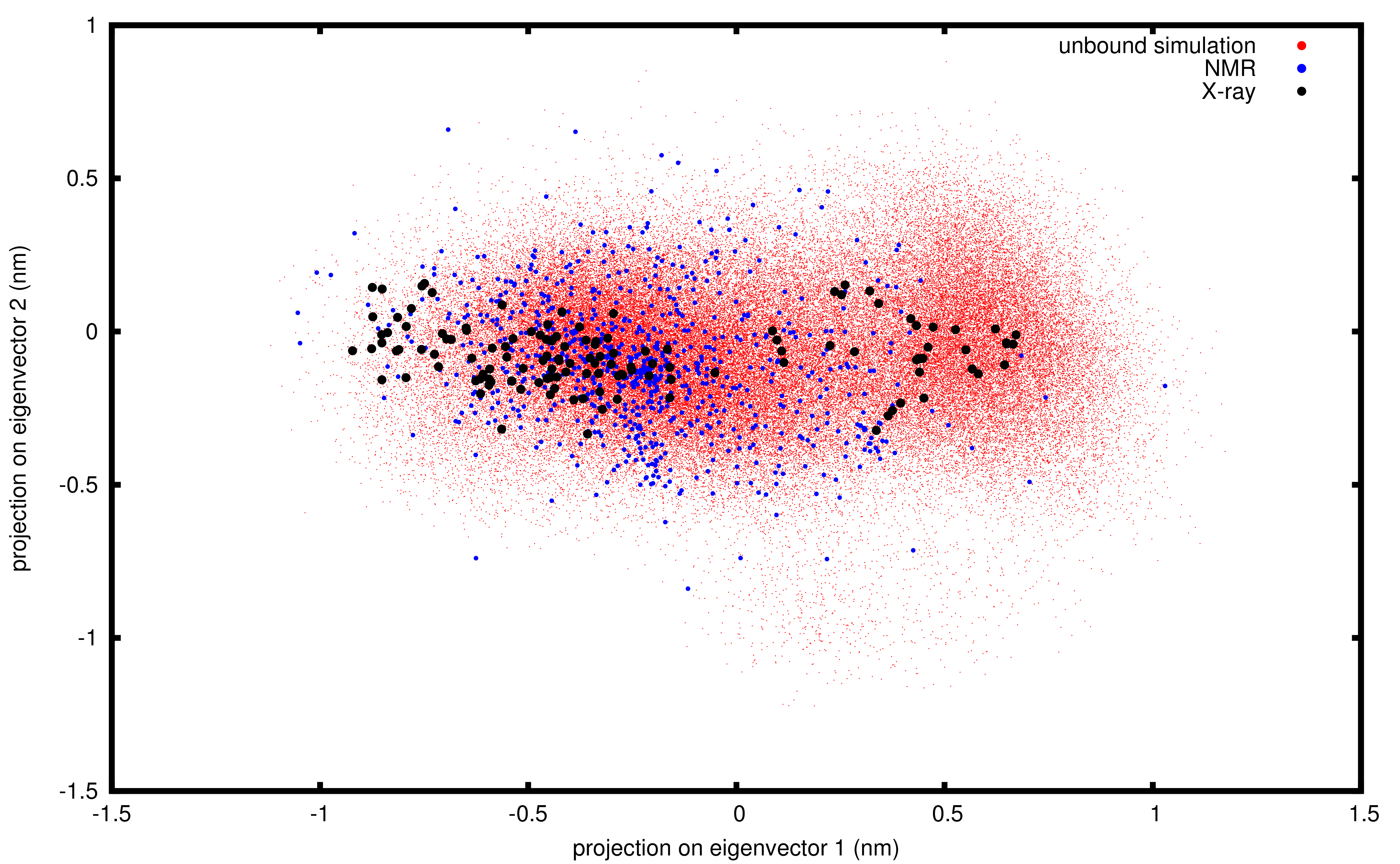

Supplement: Figure S2 — Simulation ensembles cover the same conformational space as known experimental structures. PCA projection of unbound MD simulation (starting structure 1ubi, red and a collection of experimental xray (black, 139 structures from 63 different PDB entries) and NMR (blue, 783 structures from 35 different PDB entries) structures. (TIFF) [file pcbi.1002704.s002.tif]

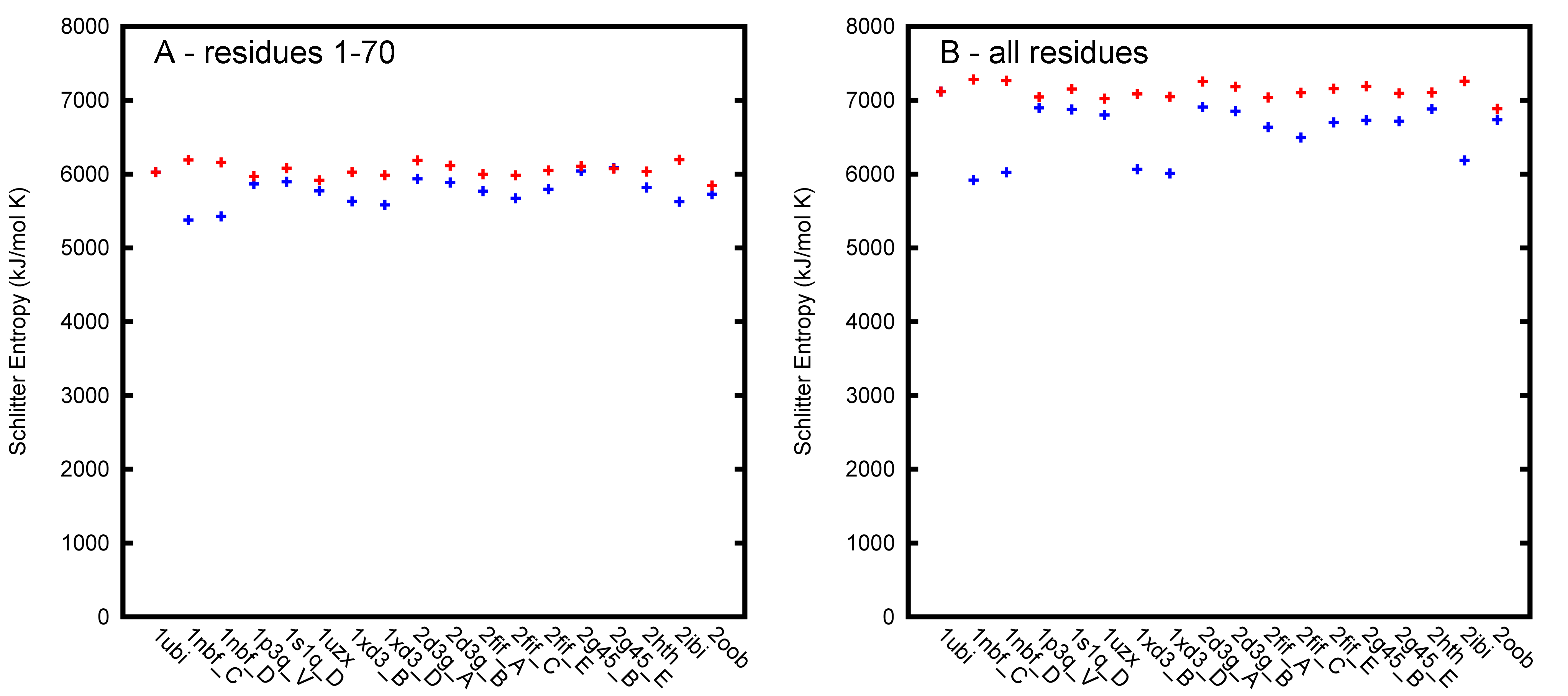

Supplement: Figure S3 — Bound ensembles show significant structural dynamics. Conformational entropy observed in unbound (blue) and bound (red) simulation ensembles estimated according to the Schlitter formula excluding (A) and including (B) the flexible C-terminus. (TIFF) [file pcbi.1002704.s003.tif]

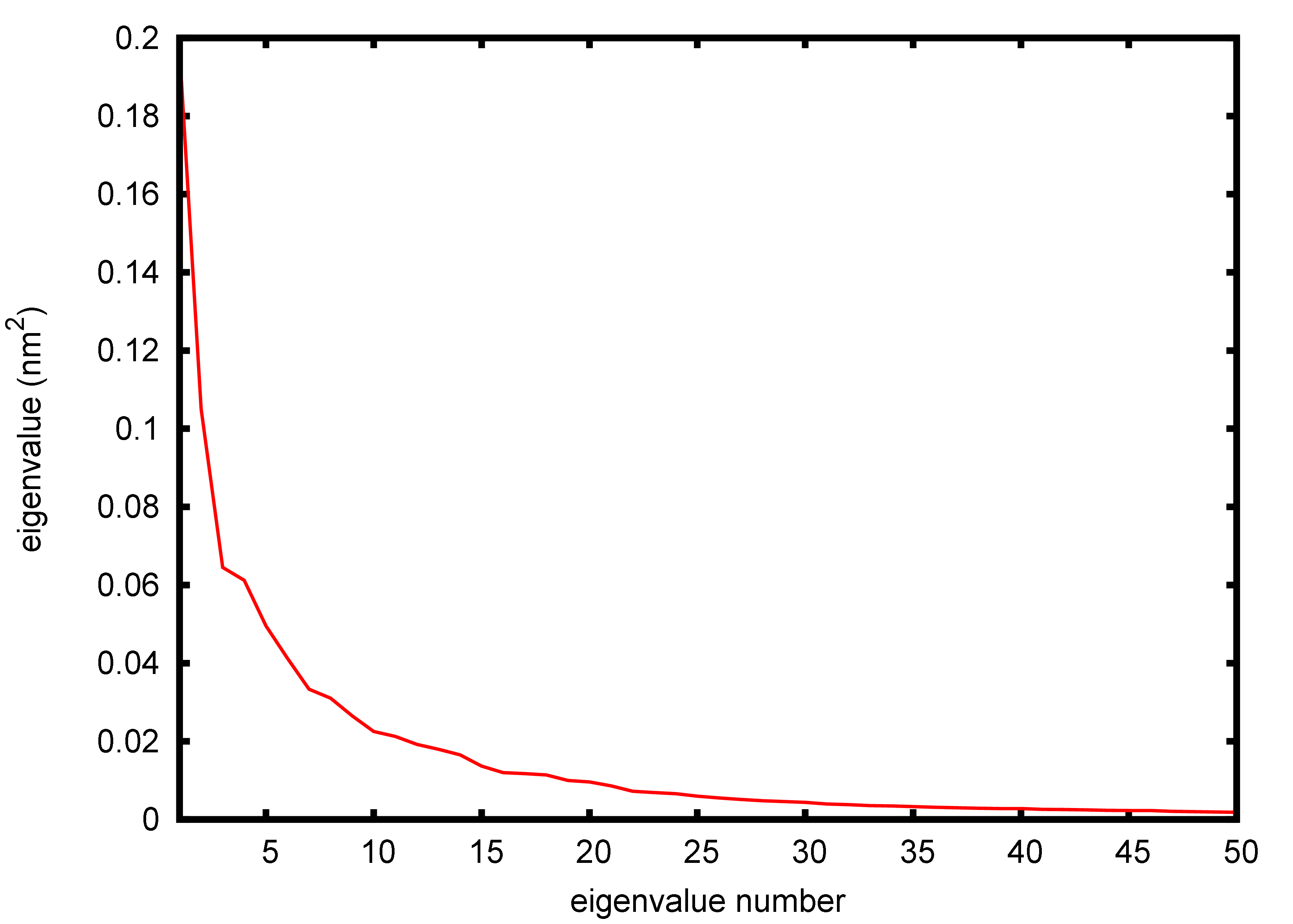

Supplement: Figure S4 — Eigenvalue spectrum for the first 50 eigenvectors of the PCA used in the main paper (backbone atoms 1–70). (TIFF) [file pcbi.1002704.s004.tif]

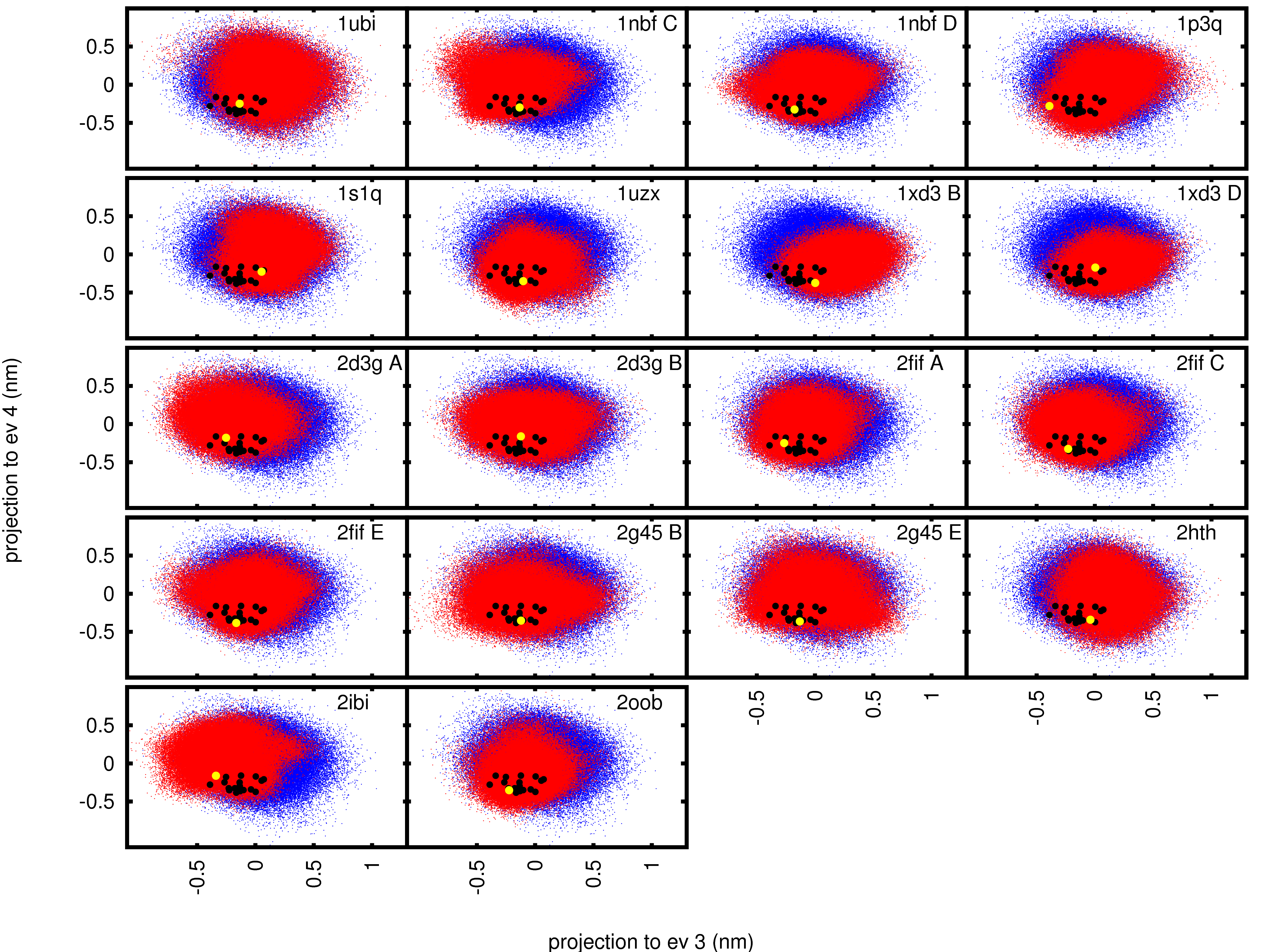

Supplement: Figure S5 — Projection to higher order eigenvectors shows no significant differences between bound and unbound ensembles. Projection to PCA-eigenvectors 3 and 4 of all simulated bound ensembles based on the backbone of ubiquitin residues 1–70. For comparison, the unbound reference ensemble is also plotted in blue, the original xray structures are marked in yellow. PDB codes for the starting structures of the simulations are in the upper right corner of each plot. Capital letters denote the chain identifier. (TIFF) [file pcbi.1002704.s005.tif]

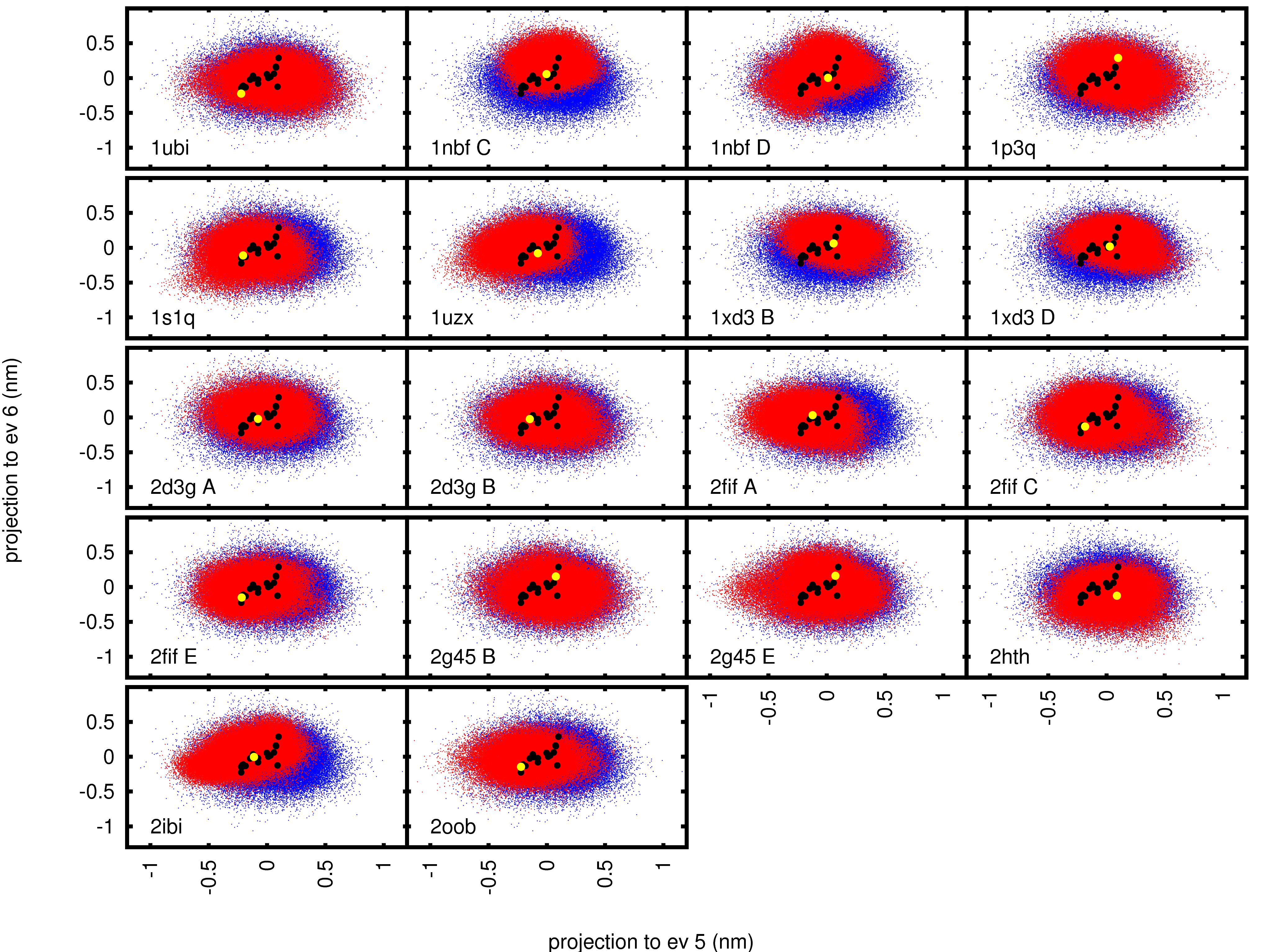

Supplement: Figure S6 — Projection to higher order eigenvectors shows no significant differences between bound and unbound ensembles. Projection to PCA-eigenvectors 5 and 6 of all simulated bound ensembles based on the backbone of ubiquitin residues 1–70. For comparison, the unbound reference ensemble is also plotted in blue, the original xray structures are marked in yellow. PDB codes for the starting structures of the simulations are in the lower left corner of each plot. Capital letters denote the chain identifier. (TIFF) [file pcbi.1002704.s006.tif]

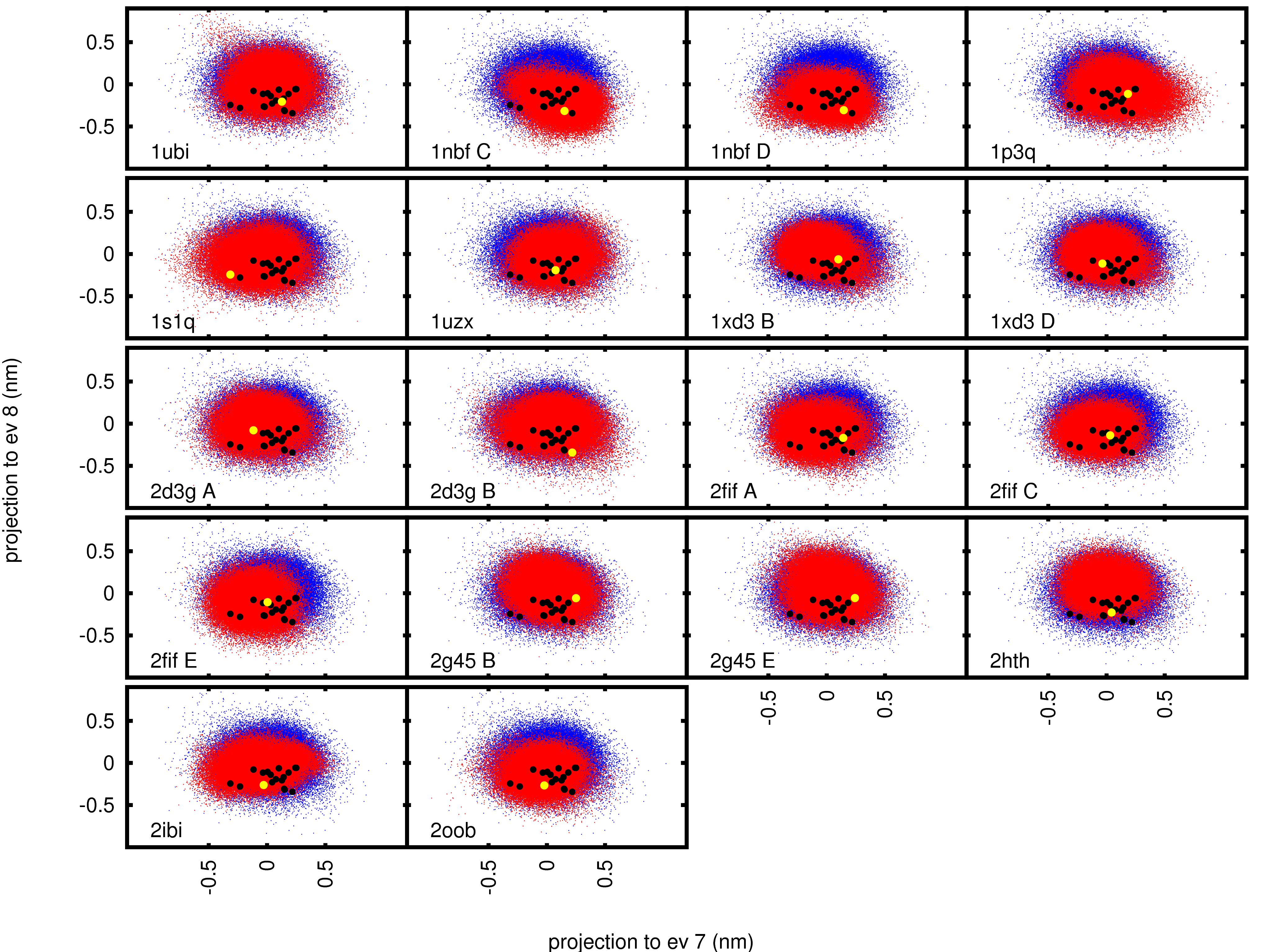

Supplement: Figure S7 — Projection to higher order eigenvectors shows no significant differences between bound and unbound ensembles. Projection to PCA-eigenvectors 7 and 8 of all simulated bound ensembles based on the backbone of ubiquitin residues 1–70. For comparison, the unbound reference ensemble is also plotted in blue, the original xray structures are marked in yellow. PDB codes for the starting structures of the simulations are in the lower left corner of each plot. Capital letters denote the chain identifier. (TIFF) [file pcbi.1002704.s007.tif]

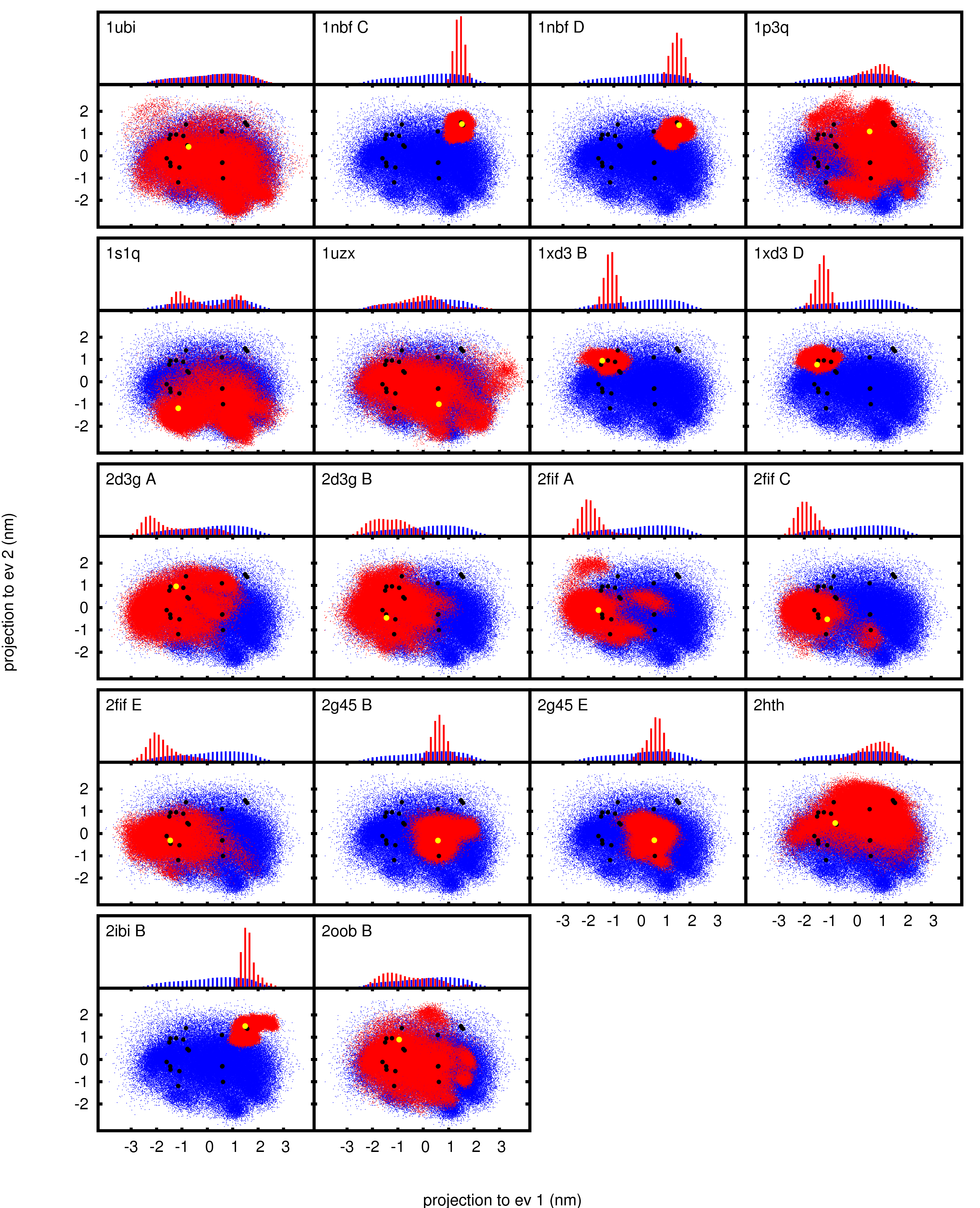

Supplement: Figure S8 — Alternative PCA including all backbone atoms of ubiquitin. Projection to the first two PCA-eigenvectors of all simulated bound ensembles based on the backbone of all ubiquitin residues (1–76). For comparison, the unbound reference ensemble is also plotted in blue, the original xray structures are marked in yellow. Histograms for the projection on the first eigenvectors are plotted above the corresponding plots. PDB codes for the starting structures of the simulations are in the upper left corner. Capital letters denote the chain identifier. (TIFF) [file pcbi.1002704.s008.tif]

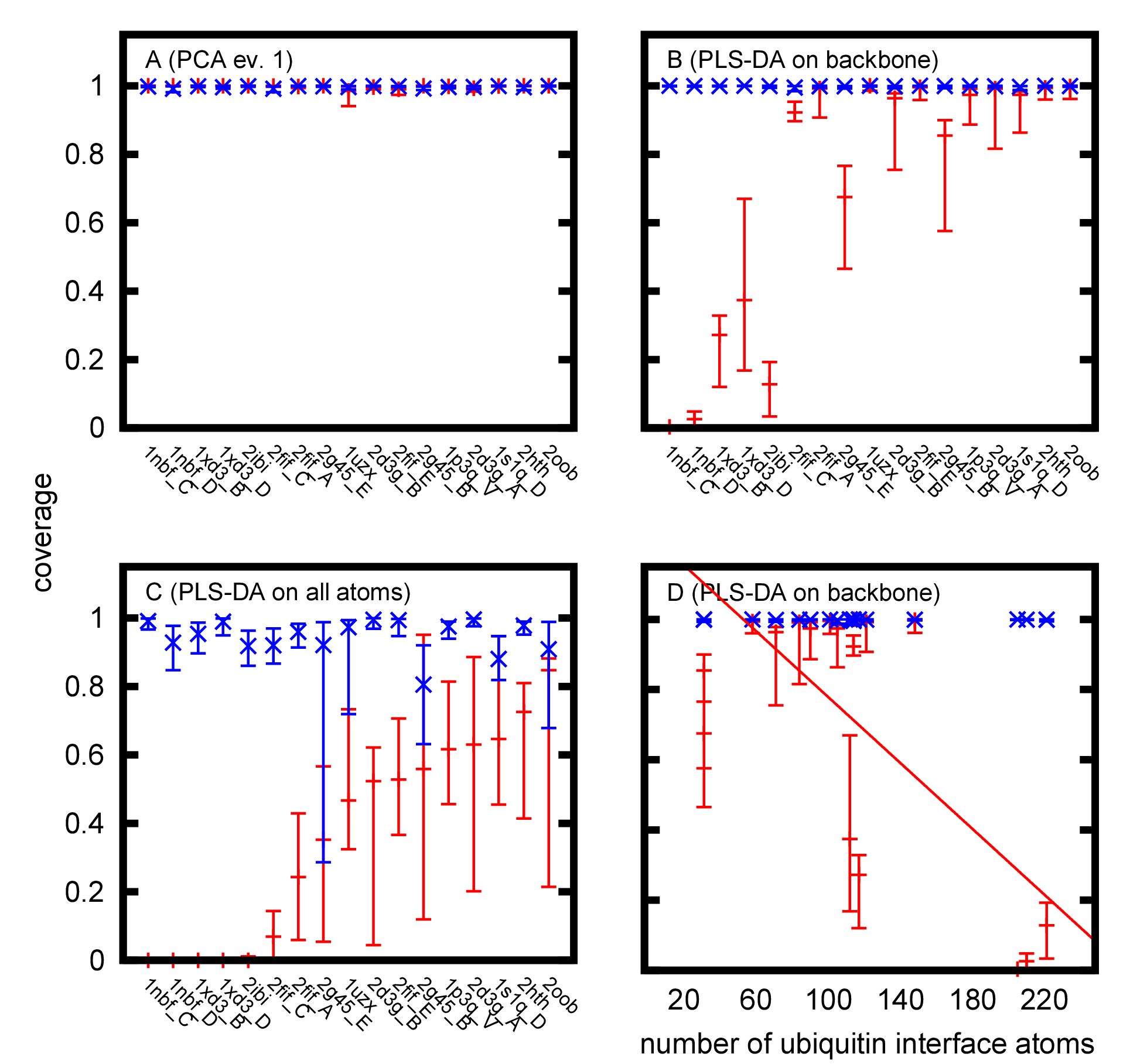

Supplement: Figure S9 — Coverage of different ensembles by the unbound reference ensemble. The histogram-coverage of bound ensembles (red) compared to coverage of unbound control ensembles (blue) after projection of the structures onto the first PCA-eigenvector (fig. 2) of backbone atoms of residues 1–76 (A), the PLS-DA difference vector of backbone atoms of residues 1–76 (B and D), and the PLS-DA difference vector of all non-hydrogen atoms of residues 1–76. Ensembles in A–C have been sorted according to the coverages displayed in C. Uncertainties have been determined using the stationary bootstrap method. (TIFF) [file pcbi.1002704.s009.tif]

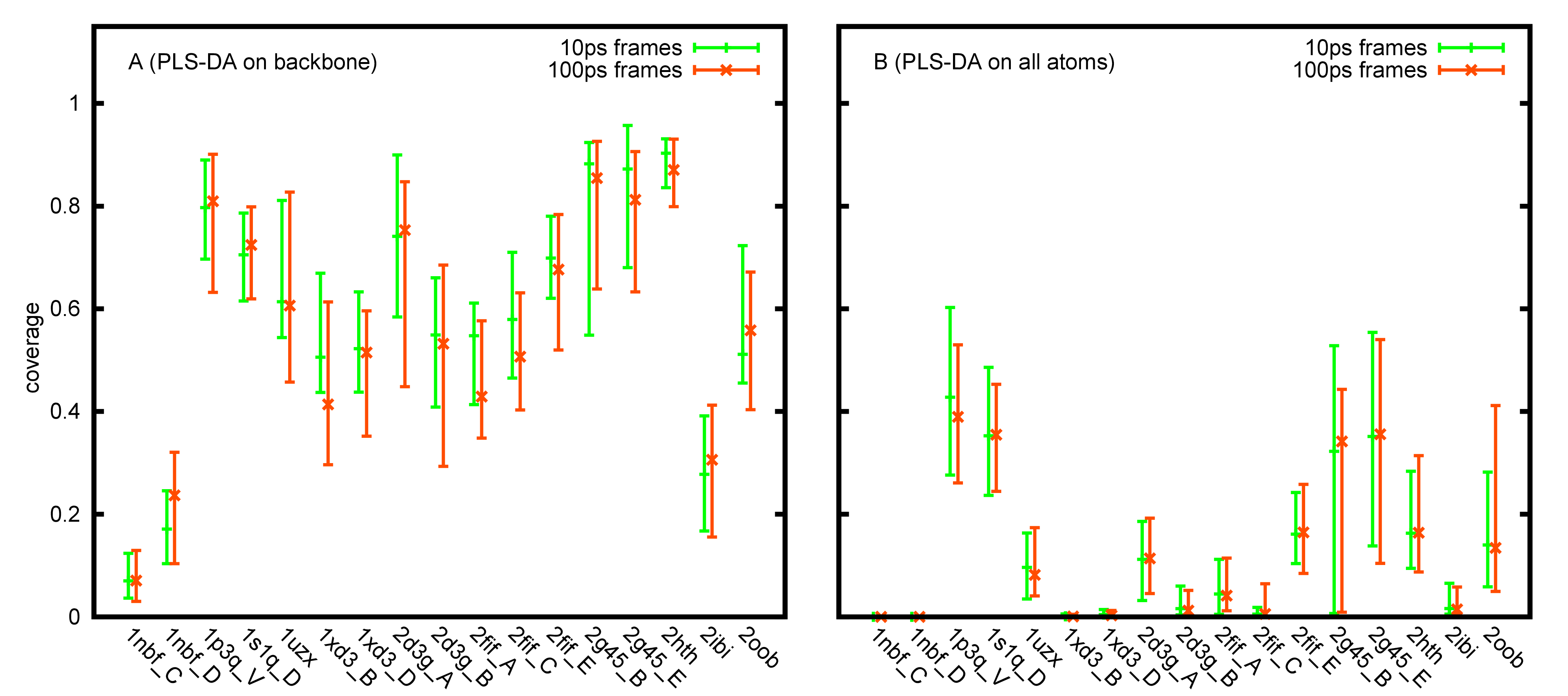

Supplement: Figure S10 — Influence of sampling on ensemble coverage. Comparison of the results illustrated in 5 B (here: A) and C (here: B) for two different sampling frequencies, (green) and (orange). (TIFF) [file pcbi.1002704.s010.tif]

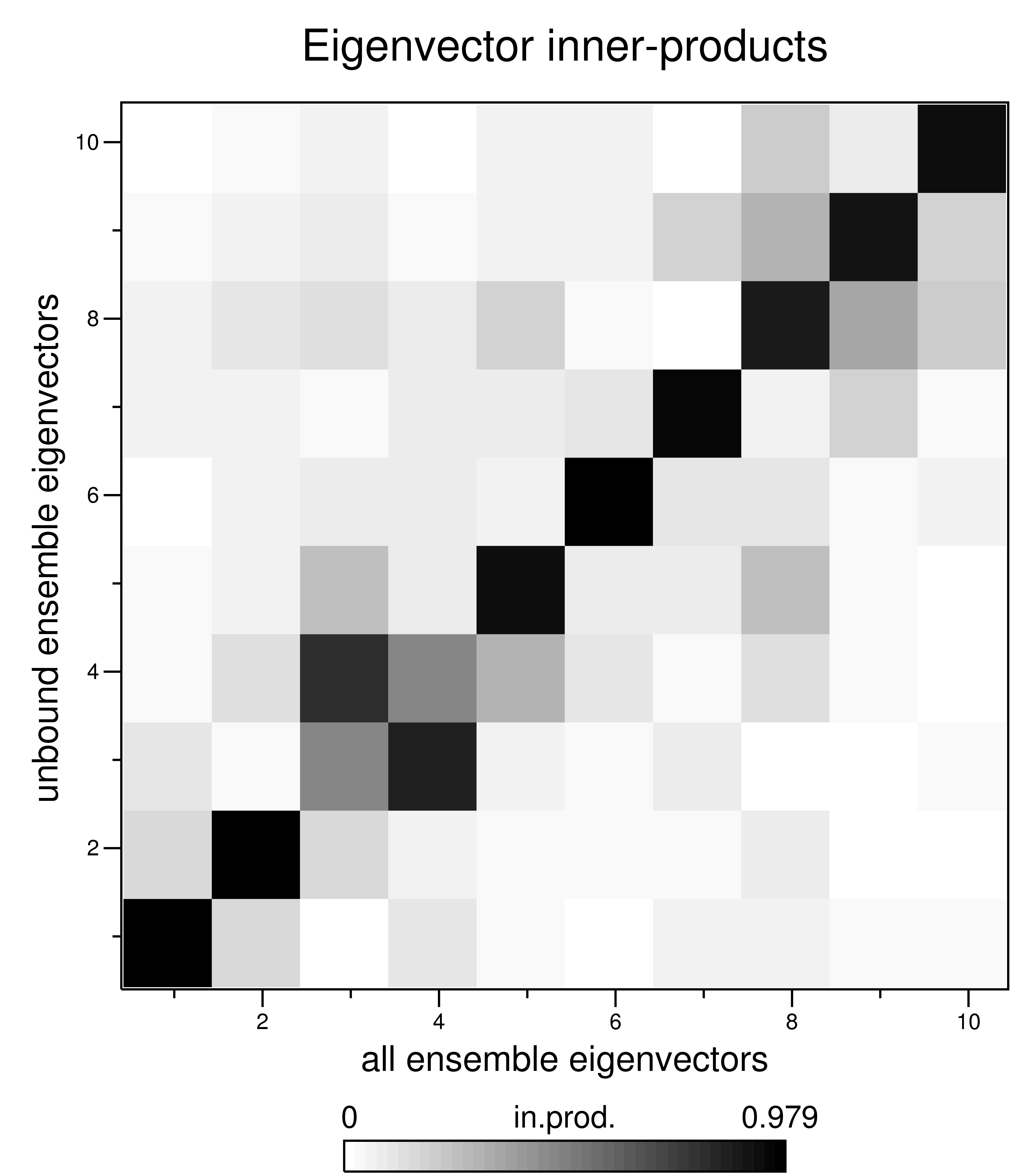

Supplement: Figure S11 — Comparison of PCA eigenvectors based on all trajectories and unbound trajectories only. Inner product calculated between the first 10 eigenvectors of both PCAs. (TIFF) [file pcbi.1002704.s011.tif]

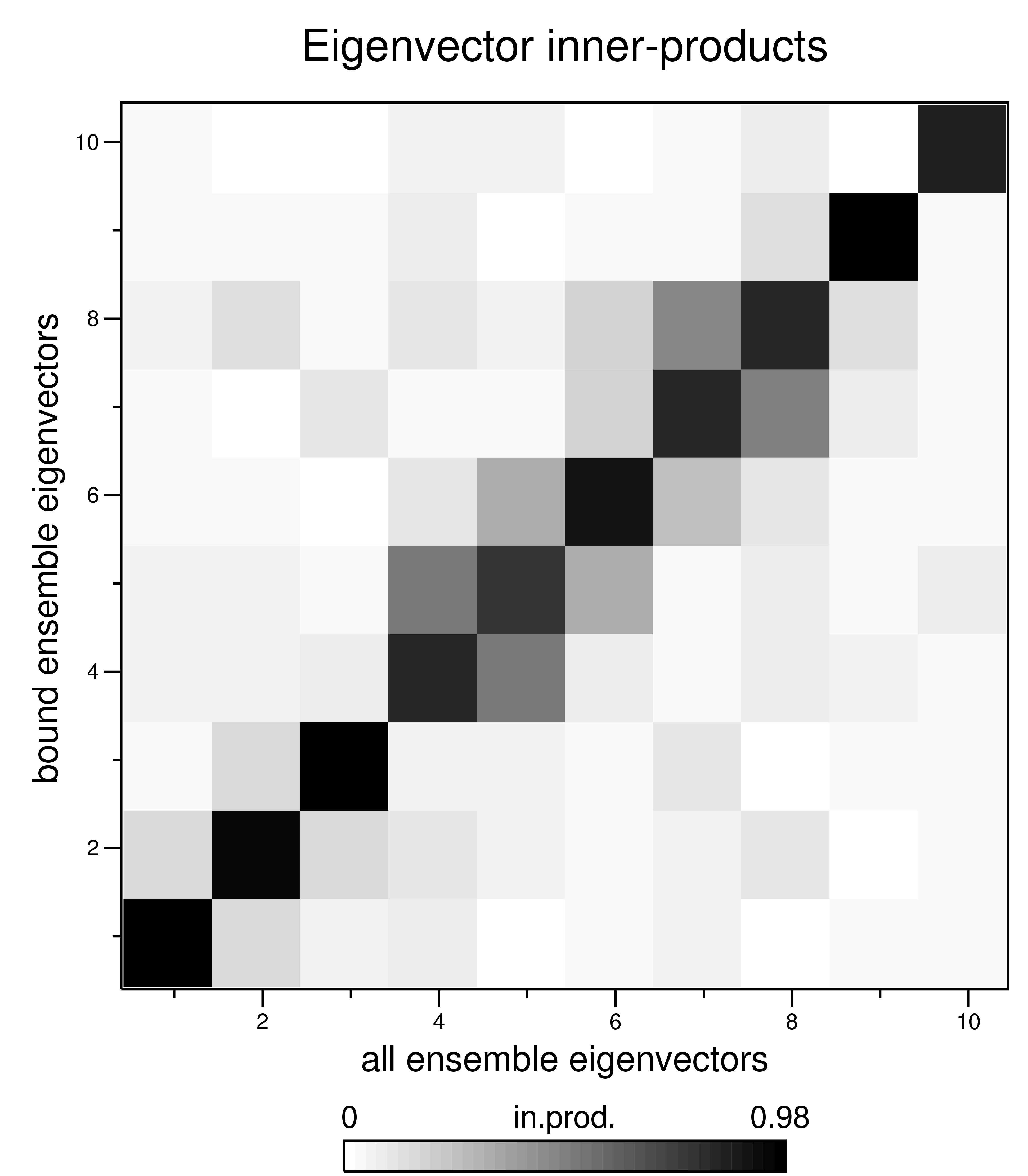

Supplement: Figure S12 — Comparison of PCA eigenvectors based on all trajectories and bound trajectories only. Inner product calculated between the first 10 eigenvectors of both PCAs. (TIFF) [file pcbi.1002704.s012.tif]

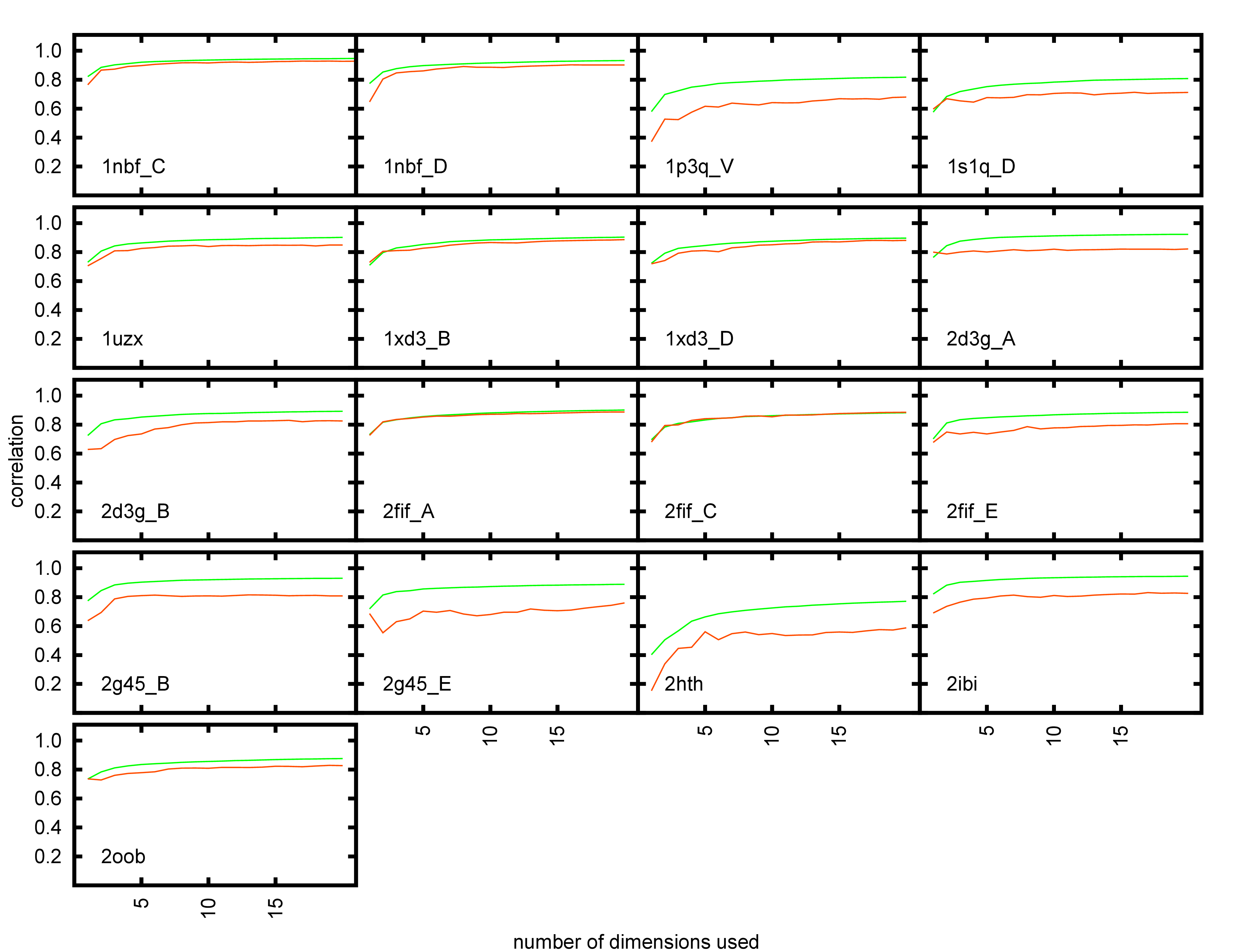

Supplement: Figure S13 — Cross correlation test of PLS-DA models. Correlation between target and model for training (green) and test (orange) set for PLS-DA between unbound and bound ensembles based on backbone atoms of residues 1–70 evaluated for different basis dimensionality. (TIFF) [file pcbi.1002704.s013.tif]
